# Mechanisms of Levetiracetam in the Control of Status Epilepticus and Epilepsy

**DOI:** 10.3389/fneur.2014.00011

**Published:** 2014-01-31

**Authors:** Laxmikant S. Deshpande, Robert J. DeLorenzo

**Affiliations:** ^1^Department of Neurology, Virginia Commonwealth University, Richmond, VA, USA; ^2^Department of Pharmacology and Toxicology, Virginia Commonwealth University, Richmond, VA, USA; ^3^Department of Biochemistry, Virginia Commonwealth University, Richmond, VA, USA

**Keywords:** levetiracetam, calcium homeostasis, status epilepticus, anti-epileptic, mechanisms

## Abstract

Status epilepticus (SE) is a major clinical emergency that is associated with high mortality and morbidity. SE causes significant neuronal injury and survivors are at a greater risk of developing acquired epilepsy and other neurological morbidities, including depression and cognitive deficits. Benzodiazepines and some anticonvulsant agents are drugs of choice for initial SE management. Despite their effectiveness, over 40% of SE cases are refractory to the initial treatment with two or more medications. Thus, there is an unmet need of developing newer anti-SE drugs. Levetiracetam (LEV) is a widely prescribed anti-epileptic drug that has been reported to be used in SE cases, especially in benzodiazepine-resistant SE or where phenytoin cannot be used due to allergic side-effects. Levetiracetam’s non-classical anti-epileptic mechanisms of action, favorable pharmacokinetic profile, general lack of central depressant effects, and lower incidence of drug interactions contribute to its use in SE management. This review will focus on LEV’s unique mechanism of action that makes it a viable candidate for SE treatment.

## Status Epilepticus: Definition, Causes and Consequences

Status epilepticus (SE) is a neurological emergency associated with a significant morbidity and mortality (1). It is defined as continuous seizure activity lasting greater than 30 min or intermittent seizures without regaining consciousness lasting for 30 min or longer (2). An operational definition of SE has also been proposed that suggests any seizures lasting more than 5 min to be considered SE and immediate steps taken to stop it to limit further morbidity and mortality (3). SE affects approximately 200,000 people annually and accounts for as many as 55,000 deaths per year in the United States alone (1). The economic burden of SE is also high with SE patients having 30–60% higher reimbursements than patients admitted for other acute health problems, including acute myocardial infarction or congestive heart failure (4). SE can be caused by acute symptomatic processes such as metabolic disturbances (for example, electrolyte imbalance, renal failure, and sepsis), CNS infection, stroke, head trauma, drug toxicity, and hypoxia (5–7). Chronic symptomatic processes that cause SE include pre-existing epilepsy or the discontinuation of anti-epileptic drugs, chronic ethanol abuse and withdrawal, and remote processes such as CNS tumors or stroke (5–7). SE can be convulsive or non-convulsive, and under both situations SE can cause significant brain damage particularly in the limbic system (8, 9). SE patients are at a higher risk of developing acquired epilepsy (10, 11). About 12–30% of adults with a new diagnosis of epilepsy first present in SE (10, 11). Further, survivors of SE suffer from other neurological problems including depression, cognitive deficits, and suicidal ideations (12).

## Treatment of SE

It is extremely important to recognize and control SE since prolonged SE can quickly develop into refractory SE, which is very difficult to treat (13). In addition, prompt SE treatment is essential to prevent mortality and the progressive brain damage that produces neurological morbidities. Treatment of SE (14) begins with medical stabilization of the patient with an initial focus on respiratory and circulatory stabilization. Further evaluations are then made looking for underlying causes of SE (metabolic disturbances, infections, etc.) and treatments are provided to correct them. Following these emergency stabilizations of the patient’s physiological status, treatment of SE is rapidly initiated using currently accepted first line drugs for stopping SE. This usually includes immediate treatment with benzodiazepines such as midazolam, diazepam, or lorazepam. The second-line of drugs to control SE include fosphenytoin, phenytoin, phenobarbital, and valproic acid. Despite the effectiveness of benzodiazepines and other anticonvulsant drugs in treating seizures, prolonged SE becomes refractory to treatment with currently available anticonvulsant agents treatment in over 40% of SE cases becoming refractory to the initial treatment with two or more medications (13). Clinical trials have shown that patients treated within 20 min of SE had better prognoses than those who did not respond within 20 min (15). However, epidemiological studies have shown that time to seizure treatment varies broadly with only about 41% of all patients receiving their first anti-epileptic drug within 30 min (16). In addition, termination of SE with benzodiazepines or phenytoin was effective in 80% of patients when administered within 30 min of seizure onset, but this effectiveness decreased to less than 40% when treatment was initiated several hours after seizure onset (17). In such a scenario, the treatment options become extremely limited to drugs such as pentobarbital, midazolam, or propofol. Topiramate and ketamine are used as additive agents to benzodiazepines and first line drugs to control refractory SE (18). However drug interactions, side-effects, pharmacoresistance, CNS depression, all add to the medical complexity of treating SE effectively and highlight the need to develop additional agents to treat SE. Thus, there is an unmet need of developing newer anti-SE drugs.

## LEV for the Treatment of SE

Levetiracetam (LEV) [(*S*)-α-ethyl-2-oxo-1-pyrrolidine acetamide] is a broad-spectrum anti-epileptic drug that was approved by the US Food and Drug Administration in 1999 and has quickly become one of the widely prescribed drugs for the treatment of partial and generalized epilepsy. While it is structurally unrelated to other anti-epileptic drugs, it is structurally related to nootropic agent piracetam. Levetiracetam is not considered a substrate for multi-drug transporters (19). The multi-drug transporter proteins are thought to be responsible for altering drug concentrations at the site of action by affecting drug uptake or increasing transport of drug cleaving enzymes. Increased expression of multi-drug transporter proteins is hypothesized to be a major mechanism for developing pharmacoresistance (20). This could explain the low probability of pharmacoresistance for LEV, despite daily chronic intake of the medication. In addition, minimal drug interactions, fewer side-effects, and broad-spectrum efficacy have all contributed to LEV’s ever widening use for the treatment of seizures. These characteristics make LEV a strong candidate for second-line treatment of SE, especially in patients with refractory SE and where use of phenytoin is deemed inappropriate due to allergic side-effects (21). With the recent introduction of an intravenous preparation of LEV, there has been considerable interest in the use of LEV for the treatment of SE (22), although LEV is not approved for this indication. There are recent studies and review articles that discuss the use of LEV in the management of SE (18, 21, 23–28). The rest of this article will mainly focus on the molecular targets and unique mechanism of actions of LEV that makes it such an attractive drug candidate for not only the treatment of SE, but also other neurological disorders such as Huntington’s chorea (29), Tardive dyskinesia (30), Tourette syndrome (31), anxiety disorders (32), traumatic brain injury and stroke (33), amongst others.

## Unique Anticonvulsant Property of LEV

Currently, little is known regarding the mechanism underlying LEV’s anti-epileptic action. The discovery of LEV’s anticonvulsant activity is unique. It was devoid of anticonvulsant activity in the acute maximal electroshock seizure test and in the maximal chemoconvulsive seizure test in pre-clinical assays (34). However, a potent protection was observed against partial epileptic seizure activity induced by pilocarpine and kainic acid (34). It also exhibited anticonvulsant activity against kindled seizures and in the Strasbourg genetic absence epilepsy rats (35). Studies attempting to elucidate LEV’s anticonvulsant action revealed a unique profile of mechanisms (36). Surprisingly, it did not exhibit the classical action in that LEV had no effect on voltage-dependent Na^+^ channels, GABAergic transmission, or affinity for either GABAergic or glutamatergic receptors (37). These represent the most common mechanisms of action for the vast majority of anti-epileptic drugs. In light of these studies, multiple laboratories focused on elucidating the molecular mechanisms that make LEV a potent anti-epileptic and SE drug. The following sections highlight the unique properties of LEV as an anticonvulsant agent.

## Effects of LEV on Neurotransmitter Release

Research has revealed several unique mechanisms for the anticonvulsant effects of LEV. Levetiracetam has been shown to affect GABA turnover in the striatum and decrease levels of the amino acid taurine, a low affinity agonist for GABA_A_ receptors, in the hippocampus with no effect in other amino acids (38). In addition, LEV removed the Zn^2+^-induced suppression of GABA_A_-mediated presynaptic inhibition, resulting in a presynaptic decrease in glutamate mediated excitatory transmission (39). Other reports have also suggested that the mechanisms of the anti-epileptic and neuroprotective actions of LEV seem to be mediated, at least in part, through the combination of inhibitory effects on depolarization-induced and Ca^2+^-induced Ca^2+^ release-associated neurotransmitter releases (40). Effects of LEV on Ca^2+^ channels have been widely studied (41, 42). Levetiracetam is also reported to modulate the presynaptic P/Q-type voltage-dependent calcium (Ca^2+^) channel to reduce glutamate release in the dentate gyrus, the area of the hippocampus that regulates seizure activities (43). Similarly, LEV has been reported to inhibit neurotransmitter release via intracellular inhibition of presynaptic Ca^2+^ channels (44).

## Levetiracetam and SV2A

Synaptic vesicle protein 2 (SV2) is a 12 trans-membrane integral protein present at all synaptic sites. It consists of three isoforms, 2A, 2B, and 2C. The SV2A isoform is most widely distributed, 2B is brain specific, and 2C is the minor brain isoform. SV2 proteins have been proposed to act as transporters of common constituent of the vesicles, such as Ca^2+^ or ATP (45). SV2A has also been shown to interact with the presynaptic protein synaptotagmin, which is considered the Ca^2+^ sensor for regulation of Ca^2+^-dependent exocytosis of synaptic vesicles (46). SV2A is involved in controlling exocytosis of neurotransmitter-containing vesicles (47). SV2A is not essential for synaptic transmission, but SV2A knockout mice exhibit seizures (48). Thus, SV2A ligands could protect against seizures through effects on synaptic release mechanisms. Indeed, SV2 has been identified as the likely target for LEV. Studies have shown that the brain distribution of the LEV-binding site, as revealed by autoradiography, matches the equivalent distribution of SV2A as determined by immunocytochemistry (45, 49). Elegant studies have shown that SV2A is indeed the binding site for LEV in the brain (50, 51). Thus, LEV’s interaction with SV2A is a leading mechanism of its anti-epileptic action.

## Levetiracetam and Ca^2+^ Signaling

Ca^2+^ ions are major second messenger molecules that play a role in plethora of biological functions including neuronal excitability and synaptic plasticity (6, 52). Ca^2+^ levels are therefore tightly regulated to attain the high signal-to-noise ratio in cellular communications. Disturbances in Ca^2+^ homeostatic mechanisms resulting in elevated intracellular Ca^2+^ levels have been reported in multiple neurological disorders including stroke, movement disorders, and seizure pathologies (6, 52). Incessant Ca^2+^ entry into the neurons via the NMDA receptors during SE and persistent leak of Ca^2+^ from intracellular Ca^2+^ stores have now been firmly established in SE induced epilepsy (6, 52). Laboratory research has shown that blocking the ryanodine receptor-mediated Ca^2+^ leak from endoplasmic reticulum using dantrolene lowers the elevated Ca^2+^ post SE and prevents the development of epileptiform discharges in hippocampal neurons (53). Interestingly, LEV reduced intra-neuronal Ca^2+^ levels by inhibiting ryanodine and IP_3_ receptor dependent Ca^2+^ release from endoplasmic reticulum (54). The ability of LEV to modulate the two major Ca^2+^-induced Ca^2+^ release systems demonstrated an important molecular effect of this agent on a major second messenger system in neurons and could possibly contribute to its unique mechanism of action. In addition, LEV has also been shown to inhibit Ca^2+^ entry by blocking the L-type Ca^2+^ channels in hippocampal neurons of spontaneously epileptic rats (55). There are other studies that report no action of LEV on L-type Ca^2+^ channels, but LEV has been shown to be selective toward N-type Ca^2+^ channels’ freshly isolated CA1 hippocampal neurons of rats (56). Thus, the effects on Ca^2+^ entry and release pathways are an important aspect of LEV’s mechanism of action.

## Levetiracetam and Epileptogenesis

The process by which healthy brain tissue is transformed by an injury into a hyperexcitable circuit of neurons giving rise to spontaneous seizures (acquired epilepsy) is called epileptogenesis (6). This transformation includes a myriad of neuronal plasticity changes including axonal sprouting, neuronal degeneration, neurogenesis, astrocytes activation, and changes in neurotransmitter release and their receptor response (6). Major second messenger systems that are activated after brain injury are suspected as initiating and sustaining these neuroplasticity changes that underlie epileptogenesis. Role of Ca^2+^ ions in epileptogenesis is well-established. Brain injury-induced protracted alterations in Ca^2+^ homeostasis are thought to trigger changes in protein transcription and gene expression that underlie abnormal synaptic plasticity changes expressed as seizure disorders and associated behavioral abnormality. Inhibition of Ca^2+^ elevations following SE are neuroprotective and produce an anti-epileptogenic effect (53, 57). Levetiracetam has been reported to limit epileptogenesis (58, 59). This effect could partly be attributed to LEV’s effect on Ca^2+^ homeostasis, as discussed above. Thus, LEV significantly inhibited development of epileptic focus following kindling-induced epileptogenesis (59). Further, a significant inhibition of seizures even at 5 weeks following termination of LEV treatment was observed in spontaneously epileptic rats indicating that LEV possesses anti-epileptogenic properties (60). However, other studies have failed in observing LEV’s anti-epileptogenic potential, for example 5-weeks of LEV treatment did not prevent development of seizures when administered 4 h after the onset of SE with seizure termination through diazepam (61). The ability of LEV to prevent development of seizures following SE makes it an important agent for the treatment of SE. Thus, LEV has important potential as an anti-epileptogenic agent that needs further elucidation.

## Concluding Remarks

Levetiracetam is a unique anticonvulsant agent that has multiple mechanism of action that differentiates it from conventional anticonvulsant drugs. This makes it an ideal agent to add to the treatments for SE. Refractory SE is a major medical and neurological emergency associated with high morbidity and mortality. Levetiracetam offers a unique anticonvulsant treatment option to initiate for the treatment of refractory SE. Its low incidence of side-effects and sedative properties make it an ideal agent to consider in treating refractory SE. The availability of an intravenous preparation of LEV also facilitates its use in treating refractory SE. Further studies should confirm that LEV will also be a major first line drug for the treatment of SE, but at present it is not approved for this use. The unique anticonvulsant mechanisms of action of LEV make it an ideal agent to add to conventional anticonvulsant agents and to consider for the treatment of refractory SE and intractable seizure disorders.

## Conflict of Interest Statement

The authors declare that the research was conducted in the absence of any commercial or financial relationships that could be construed as a potential conflict of interest.
